# The pleural mesothelium in development and disease

**DOI:** 10.3389/fphys.2014.00284

**Published:** 2014-08-01

**Authors:** Hitesh Batra, Veena B. Antony

**Affiliations:** Division of Pulmonary, Allergy and Critical Care Medicine, Department of Medicine, University of Alabama at BirminghamBirmingham, AL, USA

**Keywords:** pleural mesothelium, pleural mesothelial cells, idiopathic pulmonary fibrosis (IPF), Wilms tumor-1 (WT1), epithelial-mesenchymal transition (EMT)

## Abstract

The pleural mesothelium, derived from the embryonic mesoderm, is formed by a metabolically active monolayer of cells that blanket the chest wall and lungs on the parietal and visceral surfaces, respectively. The pleura and lungs are formed as a result of an intricate relationship between the mesoderm and the endoderm during development. Mesenchymal signaling pathways such as Wnt/B-catenin, Bmp4, and sonic hedgehog appear to be quintessential for lung development. Pleural Mesothelial Cells (PMCs) are known to express Wilms tumor-1 (Wt1) gene and in lineage labeling studies of the developing embryo, PMCs were found to track into the lung parenchyma and undergo mesothelial-mesenchymal transition (MMT) to form α-smooth muscle actin (α-SMA)-positive cells of the mesenchyme and vasculature. There is definite evidence that mesothelial cells can differentiate and this seems to play an important role in pleural and parenchymal pathologies. Mesothelial cells can differentiate into adipocytes, chondrocytes, and osteoblasts; and have been shown to clonally generate fibroblasts and smooth muscle cells in murine models. This supports the possibility that they may also modulate lung injury-repair by re-activation of developmental programs in the adult reflecting an altered recapitulation of development, with implications for regenerative biology of the lung. In a mouse model of lung fibrosis using lineage-tracing studies, PMCs lost their polarity and cell-cell junctional complexes, migrated into lung parenchyma, and underwent phenotypic transition into myofibroblasts in response to the pro-fibrotic mediator, transforming growth factor-β1 (TGF-β1). However, intra-pleural heme-oxygenase-1 (HO-1) induction inhibited PMC migration after intra-tracheal fibrogenic injury. Intra-pleural fluorescein isothiocyanate labeled nanoparticles decorated with a surface antibody to mesothelin, a surface marker of mesothelial cells, migrate into the lung parenchyma with PMCs supporting a potential role for pleural based therapies to modulate pleural mesothelial activation and parenchymal disease progression.

## Introduction

The lung is a complex, integrated structure of airways, vasculature and interstitium, surrounded by the pleural mesothelium (Jantz and Antony, 2006). The pleural mesothelium, derived from the embryonic mesoderm, is formed by a layer of cells that blanket the chest wall and lungs on the parietal and visceral surfaces, respectively. A complex interplay of signaling pathways such as Wnt/β-catenin, Bone morphogenetic protein 4 (Bmp4), sonic hedgehog, and Fibroblast growth factor 10 (Fgf10) between the developing endoderm and mesoderm is essential for development.

Wilms tumor-1 (Wt1), a zinc finger transcription factor (Haber et al., 1990), is expressed in certain mesoderm-derived tissues including the pleura (Park et al., 1993) and is known to regulate many functional properties of the developing mesothelium (Ito et al., 2006; Jomgeow et al., 2006). It also regulates pleural mesothelial cell (PMC) plasticity. Wt1 expressing PMCs have been shown to migrate into the lung parenchyma and differentiate into subpopulations of bronchial smooth muscle cells, vascular smooth muscle cells and fibroblasts (Colvin et al., 2001; Dixit et al., 2013).

The presence of Wt1-expressing PMCs on both the pleural surface and in the lung parenchyma of patients with IPF suggests a role for crosstalk between the parenchymal lung injury and activation of the pleural mesothelium in the pathogenesis of IPF. PMCs migrate into the pulmonary parenchyma in IPF and transition into myofibroblasts suggesting the novel hypothesis that IPF is an altered, recapitulation of development, with implications for regenerative biology of the lung.

PMCs could represent a new cellular therapeutic target in a disease where we currently lack treatment modalities. Intra-pleural delivery of compounds is a lung targeted, innovative therapeutic modality that can be refined to deliver drugs, minimizing systemic toxicity.

## Development

The lungs arise from the anterior foregut endoderm via a complex and highly regulated process comprising of the embryonic stage, pseudo-glandular stage, branching morphogenesis, canalicular stage, saccular stage, and the alveolarization stage. During all stages of endodermal development, the lung mesoderm interacts with the lung endoderm to generate the various lineages within the lung (Herriges and Morrisey, 2014) and plays a central role in regulating morphogenesis of the lung, including branching, lung size, and vascular development (Que et al., 2008).

### Role of mesoderm during development

The lung mesoderm is an important source of paracrine signals such as Fgf10 and Wnt2 (Bellusci et al., 1997; Sekine et al., 1999; Weaver et al., 2000; Goss et al., 2009), which are essential for multiple processes during lung development, including the patterning of early endoderm progenitors, epithelial proliferation, and differentiation. The mature lung contains many mesodermal derivatives, including airway smooth muscle, vascular smooth muscle, endothelial and mesothelial cells, as well as multiple less well-understood cell types, such as pericytes, alveolar fibroblasts, and lipofibroblasts (Herriges and Morrisey, 2014). In lineage labeling studies of the developing embryo, PMCs were found to track into the lung parenchyma and undergo mesothelial-mesenchymal transition (MMT) to form α-smooth muscle actin (α-SMA)-positive cells of the mesenchyme and vasculature (Que et al., 2008).

### Signaling pathways

During development, Wnt/β-catenin signaling is responsible for specifying Nkx2.1+ respiratory endoderm progenitors, which mark the specification of the respiratory system in the anterior foregut endoderm (Herriges and Morrisey, 2014). This, in turn, is dependent upon active Bmp signaling that represses the transcription factor Sox2, thereby allowing for expression of Nkx2.1. Loss of Bmp signaling through inactivation of the Bmp receptors, Bmpr1a and Bmpr1b, leads to tracheal agenesis with retention of the branching region of the lungs (Domyan et al., 2011). Moreover, signaling between the developing endoderm and mesoderm appears to be essential for branching morphogenesis and the loss of Fgf10 signaling to Fgfr2 in the developing endoderm leads to disruption of branching (Sekine et al., 1999; Ohuchi et al., 2000). Fgf10 expression is regulated by several signaling pathways such as Bmp4 and sonic hedgehog (Shh), suggesting a complex interplay of signaling molecules during development (Bellusci et al., 1997; Pepicelli et al., 1998; Weaver et al., 2000).

## Wt1

The Wt1 gene encodes a 49–52 kDa protein with an N-terminal domain that is involved in protein-RNA interactions critical for its transcriptional regulatory function (Call et al., 1990).

### Wt1 in prenatal development

PMCs are known to express Wt1 gene and migrate into the lung parenchyma to form smooth muscle cells of the vascular wall, as well as other cells of the lung mesenchyme during development (Que et al., 2008; Zhou et al., 2010, 2011).

Recent cell lineage labeling studies in the developing heart provide evidence that the surface epicardial mesothelium undergoes epithelial-mesenchymal transition (EMT) and migrates into the myocardium where it differentiates into various cell types, including endothelium, smooth muscle cells, and cardiomyocytes (Mikawa and Gourdie, 1996; Dettman et al., 1998; Cai et al., 2008; Zhou et al., 2010). In addition, lineage tracing and other studies show that the serosal mesothelium of the gut also contributes the majority of vascular smooth muscle cells (Wilm et al., 2005; Kawaguchi et al., 2007). A major role of Wt1 and PMCs in mesenchymal differentiation and development was demonstrated by lineage tracing studies in the embryonic mouse (Colvin et al., 2001). PMCs were shown to readily migrate into the lung parenchyma and express α-SMA. Another recent study employing Wt1^CreERT2/+^ mice visualized Wt1+ mesothelial cell entry into the lung by live imaging, identified their progenies in subpopulations of bronchial smooth muscle cells, vascular smooth muscle cells, and desmin+ fibroblasts by lineage tagging; and demonstrated that mesothelial cell movement into the lung requires the direct action of sonic hedgehog (shh) signaling (Dixit et al., 2013). Together, these studies demonstrate that the mesothelium lining of serosal surfaces of multiple organ systems plays a critical role during development and organogenesis, supporting the possibility that they may also modulate lung injury-repair by re-activation of developmental programs in the adult.

### Wt1 in postnatal disease

Wt1 can induce a morphological transition from an epithelial phenotype to a mesenchymal phenotype (Burwell et al., 2007). It is a potent transcription factor that can function as a tumor suppressor (Zhang et al., 2003), or as an oncogene depending on the cell type (Loeb et al., 2001; Oji et al., 2002; Ueda et al., 2003). It was in fact initially discovered as a tumor suppressor gene in Wilms tumor of the kidney (Haber et al., 1990). Wt1 demonstrates tissue specific responses and is recognized to regulate TGF-β1 in the kidney. In other cell types such as hematological cells it is reported to confer oncogenic properties (Licciulli and Kissil, 2010). Wt1 is expressed in normal pleural mesothelium, and is over-expressed in malignant mesothelioma (Amin et al., 1995). However, there is limited information of the role of Wt1 in non-malignant lung diseases.

### Wt1 in idiopathic pulmonary fibrosis (IPF)

Several studies support a role for Wt1 in mesothelial-to-mesenchymal transition (MMT) (Burwell et al., 2007; Bax et al., 2011a,b). Hecker et al. have evaluated the responses of PMCs to TGF-β1, their expression of MMT markers such as e-cadherin, α-SMA, vimentin; their contractile ability using a collagen gel contraction assay (Hecker et al., 2009); and also biological functional assays such as migration and proliferation (Hecker et al., 2009). Studies indicate a role for TGF-β1 in mediating MMT (Nasreen et al., 2009). For example, treatment of mouse PMCs with TGF-β1 increased expression of the myofibroblast markers α-SMA and NADPH oxidase-4 (NOX-4) (Zolak et al., 2013). Wt1-expressing PMCs are present both on the pleural surface and in the lung parenchyma of patients with IPF suggesting their role in the pathogenesis of IPF. However, the precise relationship between Wt1 and TGF-β1 remains unclear. Also, the role of Wt1 in regulating PMC plasticity needs to be analyzed.

## Pluripotency of mesothelial cells

Although mesenchymal in origin, mesothelial cells exhibit characteristics such as a polygonal cell shape, expression of surface microvilli, epithelial cytokeratins, and tight junctions, which are typical of epithelial cells (Mutsaers, 2002). During development, endothelium and vascular smooth muscle cells of the vascular system, heart, liver and gut are derived from the differentiation of mesothelium via a process of epithelial-to-mesenchymal transition (EMT) (Munoz-Chapuli et al., 1999; Perez-Pomares et al., 2002, 2004). Increasing evidence suggests existence of a population of progenitor-like mesothelial cells with the capacity to differentiate into cells of different phenotypes (Herrick and Mutsaers, 2004). By targeting mesothelin and using a genetic lineage tracing approach, Rinkevich et al. (2012) demonstrated that embryonic and adult mesothelium represents a common lineage to trunk fibroblasts, smooth muscle cells, and vasculature (Rinkevich et al., 2012).

Recently, primary rat and human mesothelial cells, when maintained in osteogenic or adipogenic media, were shown to differentiate into osteoblast- and adipocyte-like cells via epithelial-to-mesenchymal transition as suggested by the changes in mRNA expression of these cells. This supports mesothelial cell differentiation as the potential source of different tissue types in malignant mesothelioma and other serosal pathologies (Lansley et al., 2011). The term *mesodermoma* was introduced by Donna and Betta to define neoplasms arising from undifferentiated and multipotential mesoderm (Donna and Betta, 1981).

Mesothelial cells appear to retain the ability to produce mesenchyme, including smooth muscle cells, in response to transforming growth factor-β_1_ (TGF-β_1_) and platelet derived growth factor (Wada et al., 2003; Kawaguchi et al., 2007). Moreover, mesothelial cells have been shown to adopt a myofibroblast phenotype *in vitro*, in response to TGF-β_1_ (Yang et al., 2003). Transfection of the peritoneum and pleura of rats with an adenovirus expressing TGF-β_1_induced mesothelial cells to undergo EMT with subsequent fibrotic changes (Margetts et al., 2005; Decologne et al., 2007).

The significance of EMT in malignant pleural mesothelioma (MPM) was demonstrated by Schramm et al. in their study of a retrospective cohort of 352 patients. Immunohistochemistry of a tissue microarray showed that the activation of periostin-triggered EMT is associated with the sarcomatoid histotype of malignant mesothelioma and has an impact on shorter survival of patients (Schramm et al., 2010). Periostin secretion by MPM cells has in turn been shown to be upregulated by CD26 (Komiya et al., 2014), the expression of which is increased in various cancers (Havre et al., 2008).

## Idiopathic pulmonary fibrosis (IPF)

### Sub-pleural distribution

IPF is a progressive lung disease process that appears to begin in the sub-pleural regions, and then extend centrally. This sub-pleural and basilar distribution, which is the defining feature of idiopathic pulmonary fibrosis (IPF) has not been adequately explained (King et al., 2011). However, this pattern of distribution suggests pleural involvement in the disease process.

### Myofibroblasts and fibroblastic foci

The presence of myofibroblasts in fibrobastic foci in lungs of patients with IPF is well-established. The fibroblast and myofibroblast foci secrete excessive amounts of extracellular matrix, mainly collagens, resulting in scarring and destruction of the lung architecture. The profusion of fibroblastic foci is predictive of survival in IPF patients (King et al., 2001a,b). A recent study has shown that the hallmark lesion in usual interstitial pneumonia (UIP), fibroblastic foci, on three-dimensional histo-pathological reconstruction are part of a complex and highly interconnected reticulum of fibrous tissue that extends from the pleura into the lung parenchyma (Cool et al., 2006). Neither the origin of these myofibroblasts nor the molecular mechanisms involved in the formation of fibroblastic foci have been well-defined (Phan, 2008).

While a number of cellular source(s) and progenitors of tissue myofibroblasts have been proposed, none of these hypotheses provide a indisputable explanation for the histo-pathological pattern of usual interstitial pneumonia (UIP) and its peripheral localization (Raghu et al., 2011). However, evidence suggests that abnormal recapitulation of developmental pathways and epigenetic changes may play a role in the pathogenesis of IPF (King et al., 2011). IPF is a devastating disease with an inexorable course and its pathogenesis remains unclear. There are no therapies that directly impact the disease progression or mortality, and we urgently need innovative new ideas that will impact therapeutics in this disease (King et al., 2011; Raghu et al., 2011). PMCs serve as myofibroblast progenitors in animal models of fibrosis, and therapeutic targeting of these cells may be effective as an anti-fibrotic approach (Que et al., 2008).

### Pleural mesothelial cells in IPF

PMCs are metabolically active cells (Antony, 2003; Mohammed et al., 2007), responsive to their microenvironment (Antony, 2003), and are recognized to demonstrate plasticity of their phenotype (Nasreen et al., 2009). The presence of calretinin and mesothelin (markers of mesothelial cells) (Mubarak et al., 2012) and Wt-1 expressing PMCs (Zolak et al., 2013) was recently demonstrated in parenchymal cells of explanted lung tissues from 16 patients with IPF supporting a role for PMC differentiation and their trafficking into the lung as contributors to the myofibroblast population in lung fibrosis. It is not known if the number or profusion of PMCs may be predictive of the severity and/or progression of IPF.

The Ashcroft score is a score for histo-pathological grading of pulmonary fibrosis (Hagiwara et al., 2000; Matsuoka et al., 2002; Simler et al., 2002; Murakami et al., 2006). There exists a correlation between the number of calretinin-positive cells and the degree of fibrotic change in the parenchyma, as measured by the Ashcroft score. Whether the number of calretinin-positive cells was measured as a raw number, or as percentage of the total nucleated cells seen in a photomicrograph, the correlation with the degree of fibrosis was highly significant (Mubarak et al., 2012). These studies suggest that PMCs may represent a novel cellular biomarker of disease activity, and more importantly, play a role in the tissue remodeling responses seen in patients with IPF.

Recent studies demonstrate that PMCs cells lose their polarity and cell-cell junctional complexes, migrate into lung parenchyma, and undergo phenotypic transition into myofibroblasts in response to the pro-fibrotic mediator, TGF-β1 (Nasreen et al., 2009; Mubarak et al., 2012). The transition of PMC to myofibroblasts is dependent on smad-2 signaling and knockdown of smad-2 gene by silencing small interfering RNA significantly suppresses the transition of PMCs to myofibroblasts and inhibits PMC haptotaxis (Nasreen et al., 2009). We have demonstrated in a murine model, that TGF-β1 induces PMC trafficking into the lung and differentiation into myofibroblasts. Moreover, carbon monoxide or the induction of heme oxygenase-1 (HO-1) inhibits the expression of myofibroblast markers, contractility, and haptotaxis in PMCs treated with TGF-β1. These findings support a potential role for pleural-based therapies to modulate pleural mesothelial activation and parenchymal fibrosis progression (Zolak et al., 2013).

## Pleural mesothelial cells as a potential therapeutic target

PMCs migrate into the pulmonary parenchyma in IPF and transition into myofibroblasts. This invokes a novel, alternative hypothesis for the origin and source of the myofibroblasts in the lungs of patients with IPF, and provides a rational explanation for the spatio-temporal distribution of fibrosis in IPF. PMCs could represent a new cellular therapeutic target in a disease where we currently lack treatment options. Intra-pleural delivery of compounds is a lung targeted, innovative therapeutic modality that can be refined to deliver drugs, minimizing systemic toxicity.

Direct delivery of the small molecule inhibitors to the pleura could potentially have several advantages: (Jantz and Antony, 2006) It can potentially provide a direct, high concentration of the compound to target the pro-fibrogenic activities of PMCs; (Haber et al., 1990) Intra-pleural delivery might delay systemic absorption, protecting against systemic toxicity while increasing efficacy; (Park et al., 1993) Intra-pleural delivery may result in higher, sustained levels in the Bronchoalveolar lavage (BAL) when compared with serum levels (Mubarak et al., 2012); and (Jomgeow et al., 2006) innovative, less invasive methods of accessing the pleural space such as tunneled pleural catheters could make intra-pleural delivery a viable option in patients.

**Figure 1:**
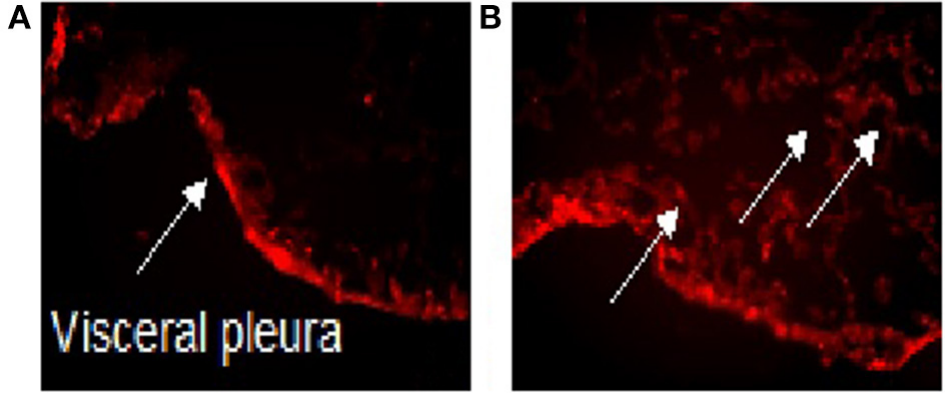
**Intra-pleural delivery of PLGA NP**. Biodegradable FITC labeled PLGA nanoparticles (which can carry therapeutic compounds conjugated to PLGA) decorated with antibody targeted to mesothelin (a PMC marker) localize to the pleural surface in control mice treated with saline **(A)**, but diffuse into the lung parenchyma in mice given intra-tracheal bleomycin **(B)**. The arrow point to FITC labeled PLGA nanoparticles. PLGA, Poly Lactic-co-Glycolic Acid; NP, Nanoparticles; FITC, Fluorescein isothiocyanate.

There are several methods that can be utilized for site-directed delivery of therapeutic agents. These include liposomal drug delivery, nanoparticle (NP) delivery of proteins, and gene therapy (Watanabe et al., 2010). Several of these methods have been utilized to target the pleura (Perez-Soler et al., 1997; Liu et al., 2006; Watanabe et al., 2010). We have demonstrated that PMCs migrate into the parenchyma (Zolak et al., 2013). Biodegradable fluorescein isothiocyanate (FITC) labeled PLGA (poly-lactic-co-glycolic acid) nanoparticles (which can carry therapeutic compounds conjugated to PLGA) decorated with antibody targeted to mesothelin (a PMC marker) localize to the pleural surface in control mice, but diffuse into the lung parenchyma in mice given intra-tracheal bleomycin (Figure 1). It appears that intra-pleural delivery of molecules to the lung is feasible, albeit, with the recognition that refinement of techniques for minimal lung injury will be required.

## Conclusion

The pleural mesothelium is a derivative of the embryonic mesoderm. Several complex pathways of interplay between the mesoderm and endoderm lead to the development of the lungs and pleura. Lineage labeling and other studies have shown the ability of the PMCs to undergo MMT to form other mesenchymal structures and have therefore established their pluripotency during development. Moreover, recent studies have shown that the plasticity of mesothelial cells is retained postnatally and gives them the ability to migrate and differentiate into other cell types. This supports the possibility that PMCs may modulate lung injury-repair by reactivation of developmental programs in the adult, reflecting an altered recapitulation of development with implications for regenerative biology of the lung. We have shown that PMCs are present in the lung parenchyma in lungs with IPF. Also, PMC migration into lung parenchyma and phenotypic transition into myofibroblasts can be induced by the pro-fibrotic mediator, TGF-β1 and is inhibited by intra-pleural heme-oxygenase-1 (HO-1). The possible role of PMCs in disease pathogenesis and/or progression of IPF makes them an attractive cellular target for potential therapeutic interventions in a disease where we currently lack treatment modalities. Intra-pleural delivery of compounds is a lung targeted, innovative therapeutic modality that can be refined to deliver drugs, thereby minimizing systemic toxicity. Intra-pleural fluorescein isothiocyanate labeled nanoparticles decorated with a surface antibody to mesothelin, a surface marker of mesothelial cells, migrate into the lung parenchyma with PMCs supporting a potential role for pleural based therapies to modulate pleural mesothelial activation and parenchymal disease progression. However, further studies are needed to develop such therapies and effective pleural-based drug delivery systems.

### Conflict of interest statement

The authors declare that the research was conducted in the absence of any commercial or financial relationships that could be construed as a potential conflict of interest.

## Abbreviations

PMCs: Pleural Mesothelial Cells (PMCs)
Wt1: Wilms tumor-1 (Wt1)
MMT: Mesothelial-mesenchymal transition (MMT)
α-SMA: α-smooth muscle actin
Bmp4: Bone morphogenetic protein 4
Shh: Sonic hedgehog
Fgf10: Fibroblast growth factor 10
IPF: Idiopathic Pulmonary Fibrosis
TGF-β1: Transforming growth factor-β1
HO-1: Heme-oxygenase-1 (HO-1)
EMT: Epithelial-mesenchymal transition
NOX-4: NADPH oxidase-4
UIP: Usual interstitial pneumonia
RNA: Ribonucleic acid
BAL: Bronchoalveolar lavage
NP: Nanoparticle
FITC: Fluorescein isothiocyanate
PLGA: Poly-lactic-co-glycolic acid.
